# Evaluated Glomerular Filtration Rate Is Associated With Non-alcoholic Fatty Liver Disease: A 5-Year Longitudinal Cohort Study in Chinese Non-obese People

**DOI:** 10.3389/fnut.2022.916704

**Published:** 2022-06-16

**Authors:** Ji Cen, Yong Han, Yufei Liu, Haofei Hu

**Affiliations:** ^1^Department of Nephrology, Hechi People’s Hospital, Hechi, China; ^2^Department of Emergency, Shenzhen Second People’s Hospital, Shenzhen, China; ^3^Department of Emergency, The First Affiliated Hospital of Shenzhen University, Shenzhen, China; ^4^Shenzhen University Health Science Center, Shenzhen, China; ^5^Department of Neurosurgery, Shenzhen Second People’s Hospital, Shenzhen, China; ^6^Department of Neurosurgery, The First Affiliated Hospital of Shenzhen University, Shenzhen, China; ^7^Department of Nephrology, Shenzhen Second People’s Hospital, Shenzhen, China; ^8^Department of Nephrology, The First Affiliated Hospital of Shenzhen University, Shenzhen, China

**Keywords:** non-alcoholic fatty liver disease, evaluated glomerular filtration rate, cohort study, non-linear, Cox proportional-hazards regression

## Abstract

**Objective:**

Evidence regarding the association between evaluated glomerular filtration rate (eGFR) and non-alcoholic fatty liver disease (NAFLD) is still limited. On that account, the purpose of our research is to survey the link of evaluated eGFR on NAFLD.

**Methods:**

This study is a retrospective cohort study. Which consecutively and non-selectively collected a total of 16,138 non-obese participants in a Chinese hospital from January 2010 to December 2014. We then used the Cox proportional-hazards regression model to explore the relationship between baseline eGFR and NAFLD risk. A Cox proportional hazards regression with cubic spline functions and smooth curve fitting (the cubic spline smoothing) was used to identify the non-linear relationship between eGFR and NAFLD. Additionally, we also performed a series of sensitivity analyses and subgroup analyses. Data had been uploaded to the DATADRYAD website.

**Results:**

The mean age of the included individuals was 43.21 ± 14.95 years old, and 8,467 (52.47%) were male. The mean baseline eGFR was 98.83 ± 22.80 mL/min per 1.73m^2^. During a median follow-up time of 35.8 months, 2,317 (14.36%) people experienced NAFLD. After adjusting covariates, the results showed that eGFR was negatively associated with incident NAFLD (HR = 0.983, 95%CI: 0.980, 0.985). There was also a non-linear relationship between eGFR and NAFLD, and the inflection point of eGFR was 103.489 mL/min per 1.73 m^2^. The effect sizes (HR) on the left and right sides of the inflection point were 0.988 (0.984, 0.991) and 0.971 (0.963, 0.979), respectively. And the sensitive analysis demonstrated the robustness of our results. Subgroup analysis showed that eGFR was more strongly associated with incident NAFLD in diastolic blood pressure (DBP) < 90 mmHg, fasting plasma glucose (FPG) ≤ 6.1 mmol/L, high-density lipoprotein cholesterol (HDL-c) < 1 mmol/L, and alanine aminotransferase (ALT) ≥ 40 U/L participants. In contrast, the weaker association was probed in those with DBP ≥ 90 mmHg, ALT < 40 U/L, FPG > 6.1 mmol/L, and HDL-c ≥ 1 mmol/L.

**Conclusion:**

This study demonstrates a negative and non-linear association between eGFR and incident NAFLD in the Chinese non-obese population. eGFR is strongly related to NAFLD when eGFR is above 103 mL/min per 1.73 m^2^. From a therapeutic perspective, it makes sense to maintain eGFR levels within the inflection point to 130 mL/min/1.73 m^2^.

## Background

Non-alcoholic fatty liver disease (NAFLD) represents a series of liver injury processes ranging from simple hepatic steatosis to non-alcoholic steatohepatitis (NASH), which can progress to cirrhosis, liver failure, and hepatocellular carcinoma (1). This is a growing public health event affecting about one in four adults worldwide (2, 3). The estimated prevalence rate of NAFLD ranges from 24.77 to 43.91% in the general population of China in recent years (4, 5). And the prevalence of NAFLD is growing worldwide (6). NAFLD is considered a hepatic component of metabolic syndrome as it is highly associated with overweight, dyslipidemia, obesity, hyperglycemia, insulin resistance, hypertension, and T2DM (7).

Clinically, obesity is closely related to NAFLD (8, 9). However, it is worth noting that many persons with a normal body mass index (BMI) are still diagnosed with NAFLD in the general population. 7.4% of non-obese adults could be diagnosed with hepatic steatosis by ultrasound in the Third National Health and Nutrition Inspection Survey of America (10). In Asia, this figure can be as high as 8–19% (11). Besides, more studies have shown that non-obese patients with NAFLD appear to be more inclined to metabolic syndrome and progress to severe liver disease more rapidly (12, 13). In addition, early detection of non-obese NAFLD can reduce the risk of diabetes and cardiovascular disease (14, 15). Therefore, identifying non-obese individuals at risk of NAFLD may still be essential. Dyslipidemia is a comorbidity of NAFLD (16). Moreover, many studies have proved that low-density lipoprotein cholesterol (LDL-c) is associated with NAFLD (17, 18). Meanwhile, a recent study suggested that elevated LDL-c levels within the normal range might play an important role in the incidence and prevalence of NAFLD (19). The rising prevalence and complexity of NAFLD in China require our persistent efforts to find new risk factors for prevention and treatment.

Estimated glomerular filtration rate (eGFR) is a more valuable and direct surrogate that reflects renal filtration function and has been widely used clinically to diagnose chronic kidney disease (CKD) and assess renal function (20). The relationship between NAFLD and CKD has attracted the attention of researchers in recent years (21). Recent studies have reported that NAFLD is related to increased incidence and prevalence of CKD (22–24). NAFLD can exacerbate insulin resistance, increase atherosclerosis-related dyslipidemia, and release various pro-inflammatory factors that may lead to renal and vascular impairment (25). In addition, NAFLD and CKD share many hepatic and renal risk factors, including diabetes, obesity, dyslipidemia, and hypertension (26). However, there are currently only fewer studies exploring the effect of CKD on NAFLD. A recent retrospective cohort study in the United States found that the incidence of NAFLD in CKD patients (eGFR < 60 mL/min/1.73 m^2^ for 90 days) was 4.4%. And the incidence of the CKD3a stage is higher than the CKD3b-5 stages (27). Another study found a high prevalence of NAFLD (56%) observed in non-diabetic CKD patients receiving hemodialysis and pre-dialysis CKD patients (28). A recent study including 2,600 Chinese patients with diabetes and NAFLD found that lower eGFR was associated with an increased likelihood of liver fibrosis (29). However, by consulting previous literature, the link between eGFR and incident NAFLD has not yet been widely explored in the health check-up population. Simultaneously, the effect of the intervention on renal function on the risk of NAFLD also needs to be further explored. Therefore, we conducted a cohort study to investigate whether the eGFR is independently associated with NAFLD in Chinese non-obese people with a normal range of LDL-c.

## Materials and Methods

### Study Design

This was a retrospective cohort study using records from a computerized database established by the Wenzhou Medical Center of Wenzhou People’s Hospital in China. The target-independent variable was the evaluated glomerular filtration rate at baseline. The outcome variable was NAFLD (dichotomous variable: 0 = non-NAFLD, 1 = NAFLD).

### Data Source

The raw data was downloaded freely from the DATADRYAD database^1^ provided by Dan-Qin et al. (30), data from: Association of low-density lipoprotein cholesterol within the normal range and NAFLD in the non-obese Chinese population: a cross-sectional and longitudinal study, Dryad, Dataset.^2^ Under Dryad’s terms of service, researchers could use this data for secondary analyses without violating authors’ rights.

### Study Population

To minimize selection bias, participants who underwent a health examination were collected non-selectively and consecutively from Wenzhou Medical Center in Wenzhou People’s Hospital. Their identity information was encoded as non-traceable codes to ensure participants’ privacy. Data were retrieved from the hospital’s electronic medical record system. The ethics committee of Wenzhou People’s Hospital approved this study. All participants have given informed consent to participate in the study (30). All methods were performed following the relevant guidelines and regulations by including a statement in the Declarations section.

**FIGURE 1:**
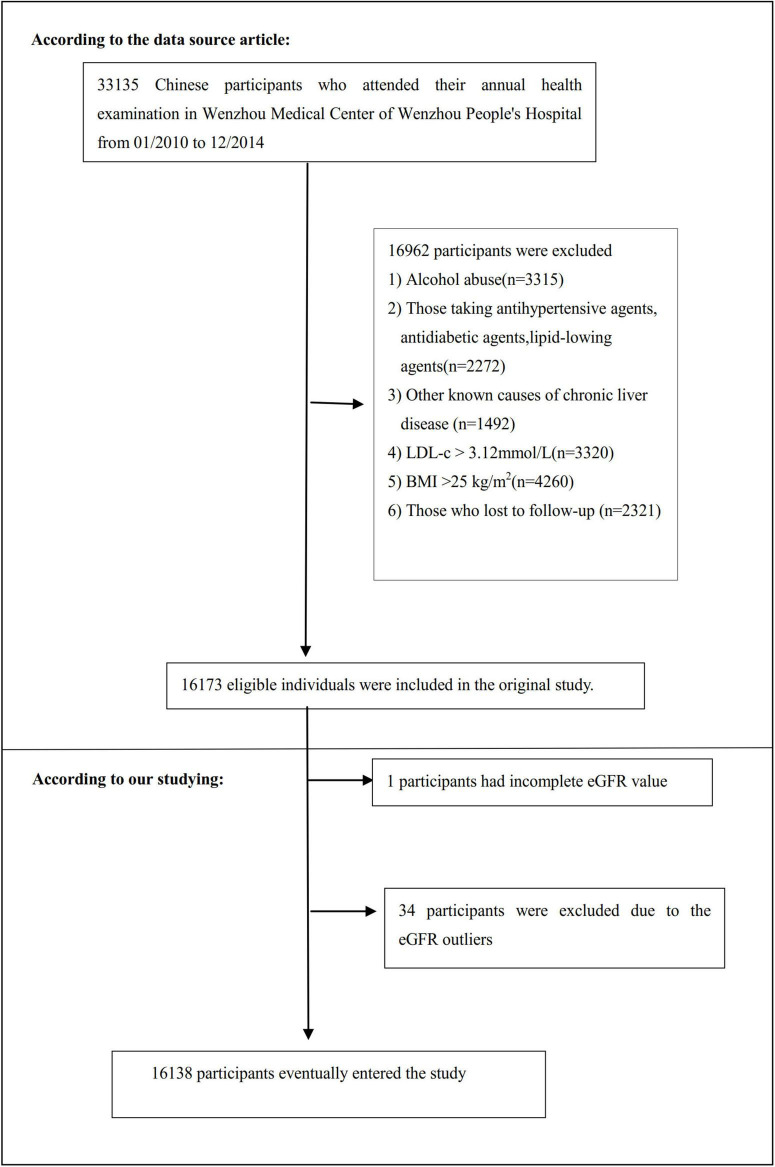
Flowchart of study participants. This figure showed the inclusion of participants. 16,173 participants were assessed for eligibility in the original study. We excluded patients with missing values of eGFR (*n* = 1), and outliers of eGFR (*n* = 34). The final analysis included 16,138 subjects in the present study.

The study initially included 33,135; thereafter, 16,997 participants were excluded. In the end, 16,138 participants were left for data analysis (see flowchart for details in Figure 1). The baseline clinical data collection’s start time and end time for these involving participants were January 2010 and December 2014, respectively. All clinical procedures in this study followed the Strobe statement (31). Inclusion criteria included: NAFLD-free Chinese individuals in the longitudinal studies who participated in a health examination from January 2010 to December 2014. Exclusion criteria included (30): (1) those with excessive alcohol consumption (per week ≥ 140 g for males and ≥ 70 g/week for females); (2) those with any known causes of chronic hepatic diseases, such as NAFLD, autoimmune hepatitis, or viral hepatitis; (3) those with BMI) ≥ 25 kg/m^2^ and LDL-c > 3.12 mmol/L; (4) those taking antihypertensive, lipid-lowering, or anti-diabetic agents; and (5) those who lost to follow-up or with missing data on total cholesterol (TC), BMI, triglyceride (TG), LDL-c, high-density lipoprotein cholesterol (HDL-c), etc.; (6) participants with incomplete eGFR; (7) those with eGFR outliers (out of the range of means plus or minus three standard deviations) (32, 33).

### Variables

#### Evaluated Glomerular Filtration Rate

We obtained the information on the evaluated glomerular filtration rate at baseline and recorded it as a continuous variable. eGFR was calculated according to the Chronic Kidney Disease Epidemiology Collaboration (CKD-EPI) equation for “Asian origin” (34). It was calculated based on age, gender, and serum creatinine (Scr) with the following formula:

Females with the concentration of Scr ≤ 0.7 mg/dL,

eGFR = 151 × (Scr/0.7)^–0^.^328^ × 0.993*^a^*^ge^;

Females with the concentration of Scr > 0.7 mg/dL,

eGFR = 151 × (Scr/0.7)^–1^.^210^ × 0.993*^a^*^ge^;

Males with the concentration of Scr ≤ 0.9 mg/dL,

eGFR = 149 × (Scr/0.9)^–0^.^415^ × 0.993*^a^*^ge^;

Males with the concentration of Scr > 0.9 mg/dL,

eGFR = 149 × (Scr/0.9)^–1^.^210^ × 0.993*^a^*^ge^;

The unit of age and Scr was year and mg/dL, respectively. This new Asian modified CKD-EPI equation may allow for more accurate GFR estimates in Chinese CKD patients in practice, especially in higher GFR populations. CKD was defined as eGFR < 60 mL/min/1.73 m^2^ for 90 days (27).

#### Outcome Measures

Our interesting outcome variable was NAFLD (dichotomous variable: 0 = non-NAFLD, 1 = NAFLD). The detailed process of measuring NAFLD was described as follows: Participants were diagnosed with NAFLD by ultrasonography, as recommended by the Chinese Liver Disease Association (35). In particular, five criteria were used to diagnose NAFLD: (1) Diffusely enhanced near-field echoes in the liver area, and gradually attenuated far-field echoes; (2) The intrahepatic cavity structure was unclear; (3) Mild to moderate hepatomegaly, with rounded edges; (4) Decreased blood flow signal in the liver; (5) The right hepatic lobe and diaphragmatic capsule were poorly visualized or incomplete (30).

Annual follow-up assessments were performed during the observation period. Liver ultrasonography was performed in a blinded (as at baseline) manner to determine the incidence of NAFLD. Participants were censored at the time of diagnosis of NAFLD or the last visit, whichever came first. The follow-up period was 5 years.

#### Covariates

Covariates were selected in our study according to our clinical experience and the previous literature. Based on the above principles, for that reason, the following variables were treated as covariates: (1) continuous variables: age, BMI, systolic blood pressure (SBP), alanine aminotransferase (ALT), diastolic blood pressure (DBP), albumin (ALB), aspartate aminotransferase (AST), globulin (GLB), γ-glutamyl transpeptidase (GGT), direct bilirubin (DBIL), LDL-c, alkaline phosphatase (ALP), uric acid (UA), total bilirubin (TB), TG, blood urea nitrogen (BUN), HDL-c, fasting plasma glucose (FPG), and TC; (2) categorical variables: gender.

Using standard methods, all the biochemical values were measured by an automated analyzer (Abbott AxSYM). A physician took a health habit inventory and medical history (30). BMI was calculated as weight in kilograms divided by height in meters square (kg/m^2^). Data was collected under standardized conditions and processed according to a uniform process. According to the World Health Organization (WHO), impaired fasting glucose (IFG) was defined as FPG of 6.1–6.9 mmol/L (36). FPG ≥ 7 mmol/L was defined as diabetes (37). ALT > 40 U/L reflected liver dysfunction (38). Hypertriglyceridemia (HTG) refers to serum TG levels ≥ 1.7 mmol/l (39). More specific details were presented in the previous reports (30, 40).

#### Statistical Analysis

Quartiles of eGFR stratified the participants. Continuous variables are expressed as mean (standard deviation) (Normal distribution) or median (range) (Non-normal distribution), and categorical variables as No. (%). We used the One-Way ANOVA test (normal distribution), the χ2 (categorical variables), or the Kruskal-Whallis H test (skewed distribution) to test for differences among different eGFR groups.

The collinearity used by the variance inflation factor (VIF) to evaluate covariates was calculated (41). VIF = 1/(1 - *R*^2^). Where *R*^2^ was the R-squared value from a linear regression equation where the dependent variable was this variable, and the independent variables were all other variables. The variables with VIF > 5 will be regarded as collinear variables and cannot be included in the multiple regression model (Supplementary Table 1).

To examine the association between eGFR and NAFLD, after collinearity screening, we constructed three models using univariate and multivariate Cox proportional-hazards regression model, including a non-adjusted model (Crude model: no covariates were adjusted), minimally adjusted model (Model I: only sociodemographic variables were adjusted, including age, gender, SBP, DBP, and BMI) and fully adjusted model (Model: covariates presented in Table 1 were adjusted, including age, SBP, sex, DBP, AST, BMI, ALB, GGT, GLB, ALP, HDL-c, DBIL, BUN, TG, ALT, FBG, TB, UA, and LDL-c). Effect sizes (HR) with 95% confidence intervals (CI) were recorded. We adjusted them when the covariances were added to the model, and the Hazard ratio (HR) changed by 10% or greater (31). Also, it referred to the results of the collinearity screening. According to the results of the collinearity screening, TC was collinear with other variables (Supplementary Table 1), so we did not finally include TC in the multivariate logistic regression equation.

**TABLE 1:** The baseline characteristics of participants.

eGFR group	Q1 (< 82.46)	Q2 (82.46–99.33)	Q3 (99.33–116.33)	Q4 (≥ 116.33)	*P*-value
Participants	4,034	4,035	4,034	4,035	
Age (years)	49.22 ± 15.98	46.78 ± 16.59	42.95 ± 12.70	33.91 ± 8.18	<0.001
**Gender**					<0.001
Male	1,320 (32.72%)	2,189 (54.25%)	2,284 (56.62%)	2,674 (66.27%)	
Female	2,714 (67.28%)	1,846 (45.75%)	1,750 (43.38%)	1,361 (33.73%)	
BMI (kg/m^2^)	22.01 ± 1.93	21.60 ± 2.04	21.16 ± 2.02	20.75 ± 1.98	<0.001
SBP (mmHg)	126.83 ± 17.33	122.01 ± 16.40	118.34 ± 15.76	115.48 ± 14.83	<0.001
DBP (mmHg)	75.50 ± 10.35	73.61 ± 10.31	71.85 ± 10.19	70.26 ± 9.80	<0.001
TC (mmol/L)	4.66 ± 0.76	4.67 ± 0.73	4.62 ± 0.73	4.55 ± 0.74	<0.001
TG (mmol/L)	1.27 (0.95–1.75)	1.12 (0.84–1.58)	1.02 (0.76–1.44)	0.93 (0.71–1.26)	<0.001
HDL-c (mmol/L)	1.40 ± 0.34	1.47 ± 0.37	1.50 ± 0.37	1.48 ± 0.37	<0.001
LDL-c (mmol/L)	2.32 ± 0.47	2.30 ± 0.46	2.25 ± 0.46	2.19 ± 0.45	<0.001
FPG (mmol/L)	5.32 ± 0.90	5.19 ± 0.79	5.09 ± 0.72	4.97 ± 0.65	<0.001
UA (μmol/L)	333.16 ± 80.63	289.71 ± 83.78	261.18 ± 78.58	234.43 ± 66.15	<0.001
Scr (μmol/L)	98.55 ± 16.15	81.27 ± 12.21	70.79 ± 11.12	60.83 ± 9.50	<0.001
eGFR (mL/min⋅1.73 m^2^)	68.78 ± 10.29	91.34 ± 4.83	107.75 ± 4.81	127.46 ± 8.32	<0.001
BUN (mmol/L)	5.05 ± 1.44	4.57 ± 1.20	4.39 ± 1.17	4.18 ± 1.13	<0.001
ALP (U/L)	76.27 ± 23.49	72.74 ± 23.77	69.38 ± 22.03	66.14 ± 21.37	<0.001
GGT (U/L)	25.00 (19.00–36.00)	22.00 (16.00–34.00)	20.00 (14.48–32.95)	18.00 (12.54–31.07)	<0.001
ALT (U/L)	18.00 (13.00–25.00)	17.00 (12.00–25.00)	16.00 (11.00–24.00)	15.00 (10.00–24.00)	<0.001
AST (U/L)	24.28 ± 10.87	23.33 ± 8.69	22.51 ± 9.18	21.43 ± 8.71	<0.001
ALB (g/L)	44.51 ± 2.84	44.47 ± 2.76	44.39 ± 2.63	44.29 ± 2.59	0.002
GLB (g/L)	29.41 ± 4.14	29.40 ± 3.88	29.62 ± 3.71	29.52 ± 3.67	0.031
TB (μmol/L)	12.79 ± 5.06	12.35 ± 4.91	11.83 ± 5.07	11.59 ± 4.80	<0.001
DBIL (μmol/L)	2.20 (1.50–3.00)	2.19 (1.50–2.98)	2.10 (1.44–2.90)	2.10 (1.45–2.98)	0.003

*Values are n (%) or mean ± SD or median (quartile).*

*BMI, Body mass index; DBP, Diastolic blood pressure; ALP, Alkaline phosphatase; SBP, Systolic blood pressure; GGT, γ-glutamyl transpeptidase; AST, Aspartate aminotransferase; TG, Triglyceride; ALB, albumin; ALT, Alanine aminotransferase; GLB, globulin; LDL-C, Low-density lipid cholesterol; BUN, Serum urea nitrogen; HDL-C, High-density lipoprotein cholesterol;Scr, Serum creatinine; TC, Total cholesterol; FPG, Fasting plasma glucose; UA, uric acid; eGFR, evaluated glomerular filtration rate; DBIL, Direct bilirubin; TB, Total bilirubin.*

To account for the non-linear relationship between eGFR and NAFLD, we also used Cox proportional hazards regression model with cubic spline functions and the smooth curve fitting to address non-linearity. Besides, the two-piecewise Cox proportional-hazards regression model was also used to further explain the non-linearity between eGFR and NAFLD (42). A log-likelihood ratio test was used to determine the most appropriate model for describing the risk associated with eGFR and NAFLD.

The subgroup analyses were performed using a stratified Cox proportional-hazards regression model across various subgroups (gender, FPG, age, BMI, HDL-c, ALT, SBP, DBP, and UA). Firstly, continuous variable age (<30, ≥30 to <40, ≥40 to <50, ≥50 to <60, ≥60 to <70, ≥70 years), BMI (<18.5, ≥18.5 to < 24, ≥ 24 kg/m^2^), FPG (≤6.1, >6.1 mmol/L), ALT (≤ 40, > 40 U/L), SBP (< 140, ≥ 140 mmHg), DBP (<90, ≥ 90 mmHg), HDL-c (<1, ≥1 mmol/L), UA (<420, ≥420 g/L) (43) were converted to a categorical variable based on the clinical cut point. Secondly, in addition to the stratification factor itself, we adjusted each stratification for all factors (age, sex, SBP, DBP, ALP, BMI, AST, ALB, ALT, GLB, GGT, HDL-c, DBIL, BUN, TG, FBG, TB, UA, and LDL-c). Lastly, tests for interaction were performed with the likelihood ratio test of models with and without interaction terms (44, 45).

The number of participants with missing data of ALP, GGT, ALT, AST, ALB, GLB, TB, DBIL, SBP, and DBP were 4,041 (25.0%), 4,043 (25.1%), 4,041 (25.0%), 4,041 (25.0%), 1,380 (8.6%), 1,380 (8.6%), 5,678 (35.2%), 7,089 (43.9%), 20 (0.1%), and 20 (0.1%), respectively. Multiple imputations were used to handle the missing data of covariants (46). The imputation model included age, sex, BMI, AST, SBP, ALB, ALT, ALP, DBP, GLB, HDL-c, DBIL, BUN, TG, UA, GGT, FBG, TC, TB, and LDL-c. Missing data analysis procedures use missing-at-random (MAR) assumptions (47).

To test the robustness of our results, we performed a series of sensitivity analyses. We converted the eGFR into a categorical variable according to the quartile and calculated the P for the trend to test the results of the eGFR as the continuous variable and explore the possibility of non-linearity. As the risk of NAFLD was obviously increased in patients with diabetes mellitus (7), IFG (48), HTG (49), CKD (28), and elevated ALT (50). Therefore, when exploring the association between eGFR and incident NAFLD in other sensitivity analyses, we excluded participants with FPG > 6.1 mmol/L, TG ≥ 1.7 mmol/L, eGFR < 60 mL/min⋅1.73 m^2^, or ALT > 40 U/L. Besides, we also used a generalized additive model (GAM) to insert the continuity covariate into the equation (model III) as a curve to ensure the robustness of the results (51). Additionally, we explored the potential for unmeasured confounding between eGFR and NAFLD risk by calculating *E*-values (52). All results were written according to the STROBE statement (31).

All the analyses were performed with the statistical software packages R (The R Foundation)^2^ and EmpowerStats^3^ (X&Y Solutions, Inc., Boston, MA). *P*-values less than 0.05 (two-sided) were considered statistically significant.

## Results

### Baseline Characteristics of Participants

The baseline characteristics of these participants were listed in Table 1. The mean age was 43.21 ± (14.95) years, and 8,467 (52.47%) were male. The mean baseline eGFR was 98.83 ± 22.80 mL/min per 1.73 m^2^. During a median follow-up time of 35.8 months, 2,317 (14.36%) people experienced NAFLD. We assigned the adults into subgroups using eGFR quartiles (<82.46, ≥82.46 to < 99.33, ≥ 99.33 to < 116.33, ≥116.33). When compared with the Q1 (<82.46) group, male, HDL-c, GLB increased significantly in the Q4 (≥116.33) group, while the opposite results were found in covariates in terms of age, female, BMI, GGT, UA, SBP, TG, AST, DBP, TC, FPG, LDL-c, Scr, ALP, ALB, BUN, TB, ALT, DBIL.

**FIGURE 2:**
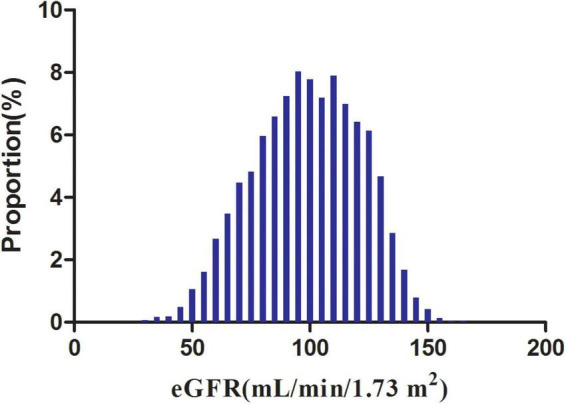
Distribution of eGFR. It presented a normal eGFR distribution while being in the range from 29.42 to 167.11 mL/min per 1.73 m^2^, with an average of 98.83 mL/min per 1.73 m^2^.

**FIGURE 3:**
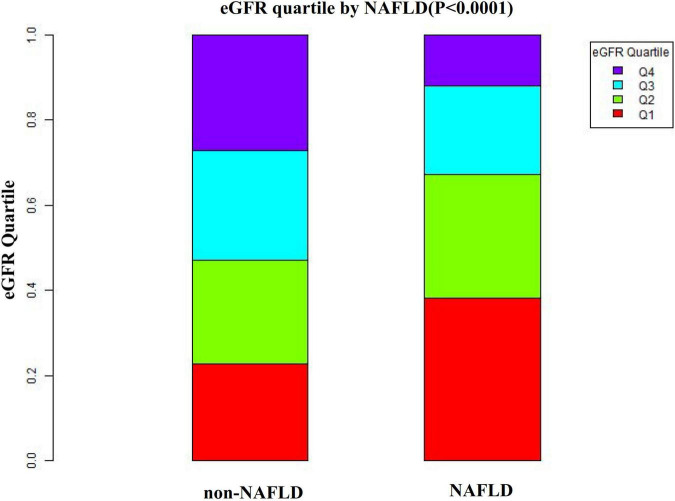
Data visualization of eGFR of all participants from the NAFLD and non-NAFLD groups. This figure indicated that the distribution level of eGFR in the NAFLD group was lower. In contrast, the eGFR level in the non-NAFLD group was relatively higher.

**FIGURE 4:**
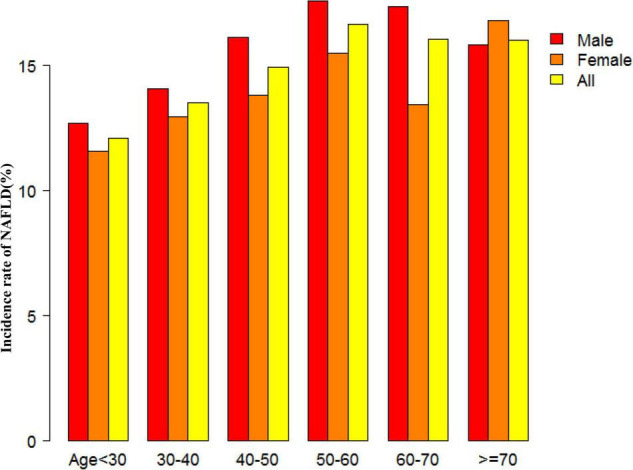
NAFLD incidence rate of age stratification by 10 intervals. This figure showed that in age stratification by 10 intervals, except for age > 70, male subjects had a higher incidence of NAFLD than female subjects no matter what age group they were in. It also found that the incidence of NAFLD increased with age, both in males (except for age > 60 years) and females (except 60–70 years old) participants.

Figure 2 showed the distribution of eGFR levels. It presented a normal distribution while being in the range from 29.42 to 167.11 mL/min per 1.73 m^2^, with an average of 98.83 mL/min per 1.73 m^2^. Participants were divided into two groups according to whether they experienced NAFLD. The eGFR levels in the two groups were shown in Figure 3 and Supplementary Figure 1. The results indicated that the distribution level of eGFR in the non-NAFLD group was higher. In contrast, the eGFR level in the NAFLD group was relatively lower. In age stratification by 10 intervals, except for age > 70, male subjects had a higher incidence of NAFLD than female subjects no matter what age group they were in Figure 4. It also found that the incidence of NAFLD increased with age, both in males (except for age > 60 years) and females (except 60–70 years old) participants.

### The Incidence Rate of Non-alcoholic Fatty Liver Disease

**FIGURE 5:**
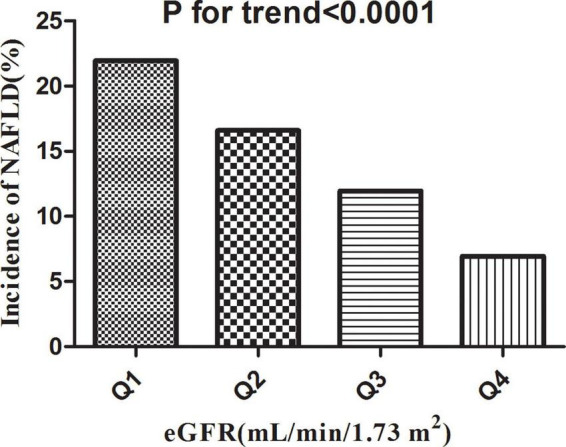
Incidence of NAFLD according to the quartiles of eGFR. Participants in the high eGFR group had a lower NAFLD incidence than the lowest eGFR group (*p* < 0.0001 for trend).

Table 2 revealed that 2,317 (14.36%) participants developed NAFLD in total during a median follow-up time of 33.77 months. The total cumulative incidence rate of all persons was 51.23 per 1,000 person-years. In particular, the cumulative incidence of the four eGFR groups were 84.57, 61.16, 42.02, and 22.69 per 1,000 person-years, respectively. The incidence rate of total NAFLD and each eGFR group was 14.36% (13.82–14.90%), 21.94% (20.66–23.22%), 16.60% (15.46–17.75%), 11.95% (10.95–12.95%), and 6.94% (6.15–7.72%), respectively. Participants with high eGFR had lower incidence rates of NAFLD compared to the group with the lowest eGFR (*p* < 0.0001 for trend) (Figure 5).

**TABLE 2:** Incidence rate of incident NAFLD.

eGFR	Participants (*n*)	NAFLD events (*n*)	Incidence rate (95% CI) (%)	Per 1,000 person-year
Total	16,138	2,317	14.36 (13.82–14.90)	51.23
Q1 (< 82.46)	4,034	885	21.94 (20.66–23.22)	84.57
Q2 (82.46–99.33)	4,035	670	16.60 (15.46–17.75)	61.16
Q3 (99.33–116.33)	4,034	482	11.95 (10.95–12.95)	42.02
Q4 (≥ 116.33)	4,035	280	6.94 (6.15–7.72)	22.69
P for trend			<0.0001	

*eGFR, evaluated glomerular filtration rate (mL/min⋅1.73 m^2^); NAFLD, non-alcoholic fatty liver disease.*

### The Results of Univariate Analyses Using Cox Proportional-Hazards Regression Model

The univariate analyses showed that NAFLD had nothing to do with ALB, GLB, and TB (all *P* > 0.05), but was positively related to age (HR = 1.006, 95%CI: 1.004–1.009), male (HR = 1.184, 95%CI: 1.091–1.285), BMI (HR = 1.815, 95%CI: 1.766–1.816), SBP (HR = 1.023, 95%CI: 1.021–1.025), DBP (HR = 1.046, 95%CI: 1.042–1.050), TC (HR = 1.298, 95%CI: 1.236–1.363), TG (HR = 1.204, 95%CI: 1.192–1.217), LDL-c (HR = 1.941, 95%CI: 1.765–2.133), ALT (HR = 1.008, 95%CI: 1.007–1.008), GGT (HR = 1.007, 95%CI: 1.007–1.008), ALP (HR = 1.009, 95%CI: 1.009–1.010), AST (HR = 1.011, 95%CI: 1.009–1.013), Scr (HR = 1.022, 95%CI: 1.020–1.023), UA (HR = 1.005, 95%CI: 1.005–1.006), FPG (HR = 1.298, 95%CI: 1.269–1.327), and negatively related to HDL-c (HR = 0.279, 95%CI: 0.246–0.317), DBIL (HR = 0.752, 95%CI: 0.727–0.778), BUN (HR = 0.931, 95%CI: 0.901–0.962), eGFR (HR = 0.980, 95%CI: 0.978–0.981) (all *P* < 0.05; Table 3).

**TABLE 3:** The results of univariate Cox proportional hazards model.

Variable	Statistics	HR (95%CI)	*P*-value
Age (years)	43.214 ± 14.951	1.006 (1.004, 1.009)	< 0.00001
**Gender**			
Female	7,671 (47.534%)	Ref.	
Male	8,467 (52.466%)	1.184 (1.091, 1.285)	0.00006
BMI (kg/m^2^)	21.380 ± 2.049	1.815 (1.766, 1.866)	< 0.00001
SBP (mmHg)	120.663 ± 16.655	1.023 (1.021, 1.025)	< 0.00001
DBP (mmHg)	72.804 ± 10.350	1.046 (1.042, 1.050)	< 0.00001
TC (mmol/L)	4.624 ± 0.743	1.298 (1.236, 1.363)	< 0.00001
TG (mmol/L)	1.301 ± 0.914	1.204 (1.192, 1.217)	< 0.00001
HDL-c (mmol/L)	1.464 ± 0.364	0.279 (0.246, 0.317)	< 0.00001
LDL-c (mmol/L)	2.264 ± 0.465	1.941 (1.765, 2.133)	< 0.00001
ALP (U/L)	71.130 ± 23.000	1.009 (1.009, 1.010)	< 0.00001
GGT (U/L)	27.983 ± 31.077	1.007 (1.007, 1.008)	< 0.00001
ALT (U/L)	19.640 ± 16.411	1.008 (1.007, 1.008)	< 0.00001
AST (U/L)	22.888 ± 9.462	1.011 (1.009, 1.013)	< 0.00001
ALB (g/L)	44.415 ± 2.708	1.011 (0.996, 1.027)	0.15575
GLB (g/L)	29.487 ± 3.856	1.005 (0.994, 1.016)	0.40472
TB (μ/L)	12.139 ± 4.982	1.002 (0.994, 1.011)	0.58034
DBIL (μ/L)	2.296 ± 1.235	0.752 (0.727, 0.778)	< 0.00001
BUN (mmol/L)	4.548 ± 1.281	0.931 (0.901, 0.962)	0.00002
Scr (μmol/L)	77.858 ± 18.730	1.022 (1.020, 1.023)	< 0.00001
UA (μmol/L)	279.618 ± 85.754	1.005 (1.005, 1.006)	< 0.00001
FPG (mmol/L)	5.143 ± 0.782	1.298 (1.269, 1.327)	< 0.00001
eGFR (mL/min⋅1.73 m^2^)	98.833 ± 22.804	0.980 (0.978, 0.981)	< 0.00001

*Values are n (%) or mean ± SD.*

*BMI, Body mass index; SBP, Systolic blood pressure; ALP, Alkaline phosphatase; DBP, Diastolic blood pressure; GGT, γ-glutamyl transpeptidase; TC, Total cholesterol; ALT, Alanine aminotransferase; Scr, Serum creatinine; LDL-C, Low-density lipid cholesterol; AST, Aspartate aminotransferase; ALB, albumin; GLB, globulin; TG, Triglyceride; UA, uric acid; HDL-C, High-density lipoprotein cholesterol; BUN, Serum urea nitrogen; FPG, Fasting plasma glucose; eGFR, evaluated glomerular filtration rate; DBIL, Direct bilirubin; TB, Total bilirubin; HR, Hazard ratios; CI, confidence interval; Ref, reference.*

**FIGURE 6:**
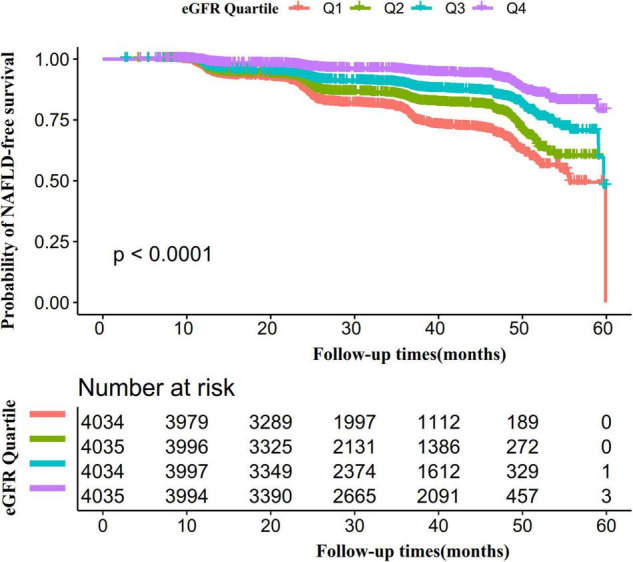
Kaplan–Meier event-free survival curve. The probability of NAFLD-free survival differed significantly between the eGFR groups (log-rank test, *p* < 0.0001). The probability of NAFLD-free survival gradually increased with increasing eGFR, suggesting that the group with the highest eGFR had the lowest risk of NAFLD.

Kaplan-Meier survival curves for NAFLD-free survival probability stratified by the eGFR group were shown in Figure 6. There were significant differences in the probability of NAFLD-free survival between the eGFR groups (log-rank test, *p* < 0.0001). The probability of NAFLD-free survival gradually increased with increasing eGFR, indicating that the group with the highest eGFR had the lowest risk of NAFLD.

### The Results of Multivariate Analyses Using Cox Proportional-Hazards Regression Model

To investigate the relationship between eGFR and incident NAFLD, the authors constructed three models using the Cox proportional-hazards regression model (Table 4). In the unadjusted model (Crude model), an increase of 1 mL/min⋅1.73 m^2^ of eGFR was connected with a 2% decrease in risk of NAFLD (HR = 0.980, 95%CI: 0.978–0.981). The results were statistically significant. In the minimally adjusted model (Model I), when we only adjusted for demographic variables, each additional mL/min⋅1.73 m^2^ of eGFR decreased by 1.5% in the risk of NAFLD (HR = 0.985, 95%CI: 0.983–0.987). The distribution of confidence intervals indicates that the relationship between eGFR and NAFLD obtained by the model was reliable. In fully adjusted model (Model II), each additional mL/min⋅1.73 m^2^ of eGFR was accompanied by a 1.7% decreases in NAFLD (HR = 0.983, 95%CI: 0.980–0.985). The results were statistically significant.

**TABLE 4:** Relationship between eGFR and the incident NAFLD in different models.

Exposure	Crude model (HR, 95%CI, *P*)	Model I (HR, 95%CI, *P*)	Model II (HR,95%CI, *P*)	Model III (HR, 95%CI, *P*)
eGFR	0.980 (0.978, 0.981) < 0.00001	0.985 (0.983, 0.987) < 0.00001	0.983 (0.980, 0.985) < 0.00001	0.989 (0.986, 0.991) < 0.00001
**eGFR Quartile**				
Q1	Ref.	Ref.	Ref.	Ref.
Q2	0.697 (0.630, 0.771) < 0.00001	0.764 (0.688, 0.848) < 0.00001	0.788 (0.708, 0.878) 0.00002	0.880 (0.789, 0.982) 0.02246
Q3	0.464 (0.415, 0.519) < 0.00001	0.606 (0.538, 0.682) < 0.00001	0.598 (0.527, 0.678) < 0.00001	0.709 (0.623, 0.808) < 0.00001
Q4	0.238 (0.208, 0.272) < 0.00001	0.364 (0.313, 0.423) < 0.00001	0.359 (0.304, 0.423) < 0.00001	0.522 (0.441, 0.618) < 0.00001
P for trend	< 0.00001	<0.00001	< 0.00001	<0.00001

*Crude model: we did not adjust other covariants.*

*Model I: we adjusted age, DBP, sex, BMI, SBP.*

*Model II: we adjusted age, SBP, sex, ALT, BMI, GGT, DBP, ALP, ALB, HDL-c, GLB, DBIL, AST, TB, UA, FBG, TG, BUN, LDL-c.*

*Model III: we adjusted age (smooth), sex, BMI (smooth), SBP (smooth), DBP (smooth), ALT (smooth), AST (smooth), GGT (smooth), ALP (smooth), ALB (smooth), GLB (smooth), DBIL (smooth), TB (smooth), BUN (smooth), UA (smooth), FBG (smooth), TG (smooth), HDL-c (smooth), LDL-c (smooth).*

*HR, Hazard ratios; CI, confidence interval; Ref, reference; eGFR, evaluated glomerular filtration rate (mL/min⋅1.73 m^2^); NAFLD, non-alcoholic fatty liver disease.*

### Sensitivity Analysis

To verify the robustness of our findings, a series of sensitivity analyses were addressed. We first transformed the eGFR from a continuous variable to a categorical variable (according to quartiles) and then put the categorically changed eGFR back into the regression equation. The results showed that the trends in effect sizes (HR) between groups were equidistant after transforming eGFR into a categorical variable. P for the trend was consistent with the result when eGFR was a continuous variable.

In addition, we used a GAM to insert the continuity covariate into the equation as a curve. The result of Model III in Table 4 showed this generally remained consistent with the fully adjusted model (HR = 0.989, 95%CI: 0.986–0.991, *P* < 0.00001). Besides, we generated an *E*-value to assess the sensitivity to unmeasured confounding. The *E*-value was 1.15. The *E*-value was greater than the relative risk of unmeasured confounders and eGFR, suggesting unmeasured or unknown confounders had little effect on the relationship between eGFR and incident NAFLD.

Furthermore, the authors excluded participants with FPG > 6.1 mmol/L in other sensitivity analyses. 553 (3.43%) participants considered IFG, and 338 (2.4%) considered diabetes. The results showed that after adjusting the confounding factors, eGFR was also negatively associated with NAFLD risk (HR = 0.983, 95% CI: 0.981–0.986) (Table 5). We also excluded participants with ALT > 40 U/L for sensitivity analyses. The results showed that after adjusting age, ALP, BMI, ALT, SBP, sex, DBP, ALB, HDL-c, AST, GLB, DBIL, TG, GGT, BUN, FBG, TB, UA, and LDL-c, eGFR was still negatively associated with NAFLD (HR = 0.983, 95% CI: 0.980–0.986) (Table 5). For sensitivity analyses, we also excluded persons with TG ≥ 1.7 mmol/L (HR = 0.987, 95% CI: 0.983–0.990) or eGFR < 60 mL/min⋅1.73 m^2^ (HR = 0.982, 95% CI:0.979–0.985). We still got similar results. To further confirm the stability of the results, we performed a Cox proportional hazards regression model on individuals with complete data and presented the results in Supplementary Table 2. After adjusting confounding variables, the results suggested that eGFR was also negatively associated with NAFLD (HR = 0.994, 95% CI: 0.990–0.998). The results obtained from all of the sensitivity analyses indicated the well-robustness of our findings (Table 5 and Supplementary Table 2).

**TABLE 5:** Relationship between eGFR and NAFLD in different sensitivity analyses.

Exposure	Model I (HR, 95%CI, *P*)	Model II (HR, 95%CI, *P*)	Model III (HR, 95%CI, *P*)	Model IV (HR, 95%CI, *P*)
eGFR	0.983 (0.981, 0.986) < 0.00001	0.983 (0.980, 0.986) < 0.00001	0.987 (0.983, 0.990) < 0.00001	0.982 (0.979, 0.985) < 0.00001
**eGFR (Quartile)**				
Q1	Ref.	Ref.	Ref.	Ref.
Q2	0.778 (0.693, 0.874) < 0.00001	0.786 (0.700, 0.833) < 0.00001	0.915 (0.787, 1.063) 0.24546	0.813 (0.727, 0.908) 0.00026
Q3	0.613 (0.535, 0.701) < 0.00001	0.639 (0.558, 0.731) < 0.00001	0.650 (0.543, 0.778) < 0.00001	0.613 (0.539, 0.698) < 0.00001
Q4	0.364 (0.306, 0.433) < 0.00001	0.376 (0.316, 0.448) < 0.00001	0.460 (0.368, 0.575) < 0.00001	0.362 (0.306, 0.428) < 0.00001
P for trend	<0.00001	<0.00001	<0.00001	<0.00001

*Model I was sensitivity analysis in participants without FPG > 6.1 mmol/L (N = 15,197). We adjusted age, SBP, sex, ALT, BMI, GGT, DBP, ALP, ALB, HDL-c, GLB, DBIL, AST, TB, UA, FBG, TG, BUN, LDL-c.*

*Model II was sensitivity analysis in participants without ALT > 40 U/L (N = 15,045). We adjusted age, SBP, sex, ALT, BMI, GGT, DBP, ALP, ALB, HDL-c, GLB, DBIL, AST, TB, UA, FBG, TG, BUN, LDL-c.*

*Model III was sensitivity analysis in participants without TG ≥ 1.7 mmol/L (N = 13,069). We adjusted age, SBP, sex, ALT, BMI, GGT, DBP, ALP, ALB, HDL-c, GLB, DBIL, AST, TB, UA, FBG, TG, BUN, LDL-c.*

*Model IV was sensitivity analysis in participants without eGFR < 60 mL/min⋅1.73 m^2^ (N = 15,362). We adjusted age, SBP, sex, ALT, BMI, GGT, DBP, ALP, ALB, HDL-c, GLB, DBIL, AST, TB, UA, FBG, TG, BUN, LDL-c.*

*HR, Hazard ratios; CI, confidence interval; Ref, reference; eGFR, evaluated glomerular filtration rate (mL/min⋅1.73 m^2^); NAFLD, non-alcoholic fatty liver disease.*

### The Non-linearity Addressed by Cox Proportional Hazards Regression Model With Cubic Spline Functions

**FIGURE 7:**
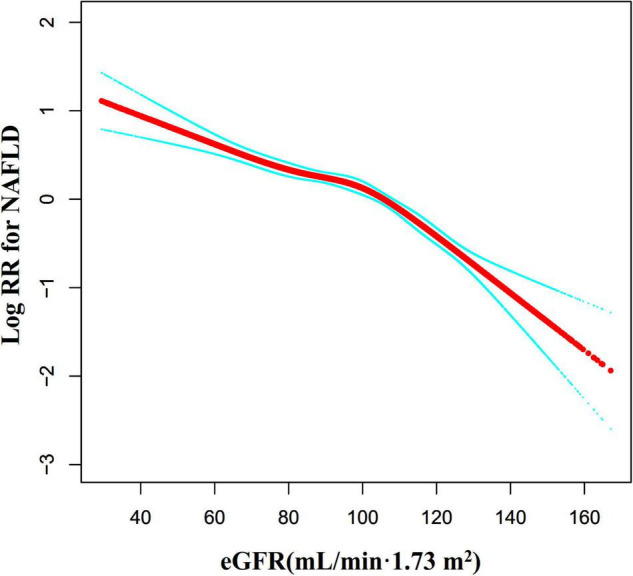
The non-linear relationship between eGFR and the risk of NAFLD. We used a Cox proportional hazards regression model with cubic spline functions to evaluate the relationship between eGFR and NAFLD risk. The result showed that the relationship between eGFR and NAFLD was non-linear, with the inflection point of eGFR being 103.489 mL/min⋅1.73 m^2^.

Through the Cox proportional hazards regression model with cubic spline functions, we observed that the association between eGFR and NAFLD was also non-linear (Figure 7). Therefore, fit the data to a piecewise Cox proportional hazards regression model to obtain two distinct slopes. We also fit the data by a standard Cox proportional hazards regression model and determine the best fit model by a log-likelihood ratio test (Table 6). In the present study, the P for the log-likelihood ratio test was < 0.001. By recursive algorithm, we first got the inflection point was 103.489 mL/min⋅1.73 m^2^ and then calculated the HR and CI on the left and right of the inflection point by two-piecewise Cox proportional-hazards regression model. On the left side of the inflection point, the HR and 95%CI were 0.988 (0.984, 0.991). On the right side of the inflection point to eGFR ≤ 130 mL/min/1.73 m^2^, the HR and 95%CI were 0.971 (0.963, 0.979). As we were known, individuals in the early phase of renal impairment can present with glomerular hyperfiltration, which is most often defined as a GFR > 130 mL/min/1.73 m^2^. Therefore, In Table 6, on the basis of eGFR > 103.489 mL/min/1.73 m^2^, we further analyzed the relationship between eGFR and NAFLD risk in the population with eGFR > 130 mL/min/1.73 m^2^. The results suggested that when eGFR > 130 mL/min/1.73 m^2^, the association between eGFR and NAFLD risk was not statistically significant (HR = 0.969, 95%CI: 0.928, 1.012, *P* = 0.1584).

**TABLE 6:** The result of the two-piecewise Cox regression model.

Incident NAFLD	Model I (HR, 95%CI, P)	Model II (HR, 95%CI, P)
Fitting model by standard Cox regression	0.986 (0.984, 0.988) < 0.0001	0.982 (0.979, 0.985) < 0.0001
**Fitting model by two-piecewise Cox regression**		
Inflection point of eGFR	103.489	103.117
≤Inflection point	0.988 (0.984, 0.991) < 0.0001	0.988 (0.984, 0.992) < 0.0001
>Inflection point, ≤ 130	0.971 (0.963, 0.979) < 0.0001	0.970 (0.962, 0.979) < 0.0001
>130	0.969 (0.928, 1.012) 0.1584	0.969 (0.928, 1.012) 0.1563
P for log-likelihood ratio test	<0.001	<0.001

*Model I: Analysis among all participants; Model II: sensitivity analysis in participants without eGFR < 60 mL/min⋅1.73 m^2^ (N = 15,362).*

*We adjusted age, SBP, sex, ALT, BMI, GGT, DBP, ALP, ALB, HDL-c, GLB, DBIL, AST, TB, UA, FBG, TG, BUN, LDL-c.*

*HR, Hazard ratios; CI, confidence interval; Ref, reference; eGFR, evaluated glomerular filtration rate (mL/min⋅1.73 m^2^); NAFLD, non-alcoholic fatty liver disease.*

**FIGURE 8:**
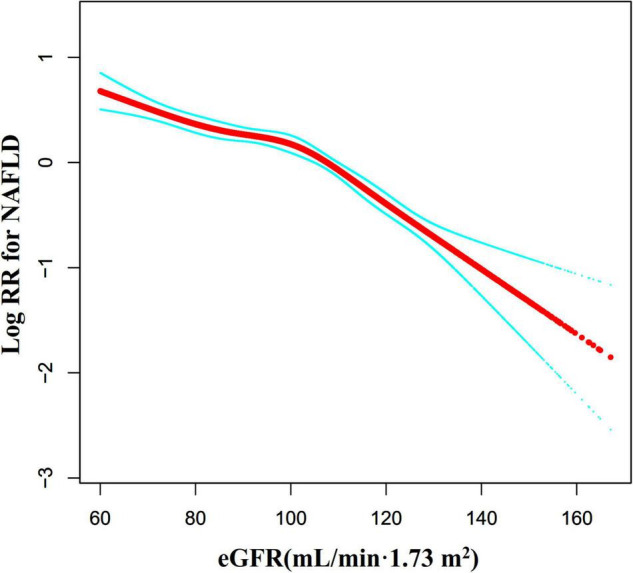
The non-linear relationship between eGFR and NAFLD risk in participants with eGFR ≥ 60 mL/min⋅1.73 m^2^. We also used a Cox proportional hazards regression model with cubic spline functions to evaluate the relationship between eGFR and NAFLD risk in participants with eGFR ≥ 60 mL/min⋅1.73 m^2^. The result showed that the relationship between eGFR and NAFLD was non-linear, with the inflection point of eGFR being 103.117 mL/min⋅1.73 m^2^.

We also used the Cox proportional hazards regression model with cubic spline functions to explore the non-linear relationship between eGFR and NAFLD in participants without eGFR < 60 mL/min⋅1.73 m^2^ for the sensitivity analysis. We found that the association between eGFR and NAFLD was still non-linear (Figure 8). The inflection point of the eGFR was 103.117 mL/min⋅1.73 m^2^. On the left side of the inflection point, the HR and 95%CI were 0.988 (0.984, 0.992). On the right side of the inflection point to eGFR ≤ 130 mL/min/1.73 m^2^, the HR and 95%CI were 0.970 (0.962, 0.979). When eGFR > 130 mL/min/1.73 m^2^, the association between eGFR and NAFLD risk was also not statistically significant (HR = 0.969, 95%CI: 0.928, 1.012, *P* = 0.1563) (Table 6).

### The Results of Subgroup Analyses

In all of the prespecified or exploratory subgroups evaluated (Table 7), there was no significant interaction in age, BMI, UA, LDL-c, gender, or SBP. In contrast, significant interactions were detected in variables such as DBP, ALT, HDL-c, and FPG.

**TABLE 7:** Effect size of eGFR on NAFLD in prespecified and exploratory subgroups.

Characteristic	No of participants	HR (95%CI)	*P*-value	P for interaction
Age, yearsv				0.1606
<30	3,109	0.980 (0.975, 0.985)	< 0.0001	
30–40	4,746	0.986 (0.982, 0.991)	< 0.0001	
40–50	3,674	0.981 (0.976, 0.986)	< 0.0001	
50–60	2,128	0.985 (0.979, 0.992)	< 0.0001	
60–70	1,109	0.976 (0.967, 0.985)	< 0.0001	
≥70	1,372	0.978 (0.969, 0.987)	< 0.0001	
Gender				0.9823
Male	8,467	0.983 (0.979, 0.986)	< 0.0001	
Female	7,671	0.983 (0.980, 0.986)	< 0.0001	
BMI (kg/m^2^)				0.6062
<18.5	1,462	0.970 (0.932, 1.009)	0.1292	
≥18.5, < 24	12,821	0.981 (0.978, 0.983)	< 0.0001	
≥ 24	1,855	0.982 (0.979, 0.986)	< 0.0001	
FPG (mmol/L)				0.0357
≤6.1	15,197	0.982 (0.979, 0.985)	< 0.0001	
>6.1	941	0.988 (0.983, 0.994)	< 0.0001	
HDL-c (mmol/L)				<0.0001
<1	1,283	0.971 (0.966, 0.976)	< 0.0001	
≥1	14,855	0.984 (0.982, 0.987)	< 0.0001	
ALT (U/L)				0.0431
≤40	15,095	0.983 (0.980, 0.986)	< 0.0001	
>40	1,043	0.977 (0.971, 0.983)	< 0.0001	
UA (μmol/L)				0.9899
<420	15,109	0.983 (0.981, 0.985)	< 0.0001	
≥420	1,029	0.983 (0.977, 0.989)	< 0.0001	
SBP (mmHg)				0.4950
<140	14,075	0.983 (0.980, 0.986)	< 0.0001	
≥140	2,063	0.984 (0.980, 0.989)	< 0.0001	
DBP (mmHg)				0.0360
<90	14,992	0.982 (0.980, 0.985)	< 0.0001	
≥90	1,146	0.988 (0.983, 0.993)	< 0.0001	
LDL-c (mmol/L)				0.2171
<2.28	7,965	0.981 (0.978, 0.985)	< 0.0001	
≥2.28	8,173	0.984 (0.981, 0.987)	< 0.0001	

*Above model adjusted for age, SBP, sex, ALT, BMI, GGT, DBP, ALP, ALB, HDL-c, GLB, DBIL, AST, TB, UA, FBG, TG, BUN, LDL-c.*

*In each case, the model is not adjusted for the stratification variable.*

*HR, Hazard ratios; CI, confidence interval; Ref, reference; eGFR, evaluated glomerular filtration rate (mL/min⋅1.73 m^2^); NAFLD, non-alcoholic fatty liver disease.*

Specifically, a stronger association between eGFR and NAFLD was observed in DBP < 90 mmHg (HR = 0.982, 95%CI: 0.980–0.985), FPG ≤ 6.1 mmol/L (HR = 0.982, 95%CI: 0.979-0.985), HDL-c < 1 mmol/L (HR = 0.971, 95%CI: 0.966–0.976), and ALT ≥ 40 U/L (HR = 0.977, 95%CI: 0.971–0.983) participants. In contrast, the weaker association was probed in those with DBP ≥ 90 mmHg (HR = 0.988, 95%CI: 0.983–0.993), ALT < 40 U/L (HR = 0.983, 95%CI: 0.980–0.986), FPG > 6.1 mmol/L (HR = 0.988, 95%CI: 0.983–0.994), and HDL-c ≥ 1 mmol/L (HR = 0.984, 95%CI: 0.982-0.987).

## Discussion

This retrospective cohort study explored the association of eGFR with NAFLD risk. Our results indicated that the increase of eGFR was associated with a significantly decreased risk of NAFLD. In addition, a threshold effect curve was found as well, and different associations of eGFR on the NAFLD were detected on both sides of the inflection point. In addition, DBP, HDL-c, ALT, and FPG were found as the potential effect modifiers to modify the relationship between eGFR and NAFLD, as significantly stronger associations were observed in DBP < 90 mmHg, FPG ≤ 6.1 mmol/L, HDL-c < 1 mmol/L, and ALT ≥ 40 U/L participants. While considerably weaker associations were detected in individuals with those with DBP ≥ 90 mmHg, ALT < 40 U/L, FPG > 6.1 mmol/L, and HDL-c ≥ 1 mmol/L.

A retrospective cohort study of 1,155,901 patients in the United States found that during a median follow-up of 4.74 years, 51,584 (4.4%) of CKD patients developed NAFLD. At the same time, they discovered that NAFLD incidence in patients with CKD 3a stage was higher than that in CKD3b-5 stage patients. However, our study found that NAFLD incidence was 14.36% after a median follow-up of 3.77 years in the population with physical examination in Chinese hospitals. By comparing the populations of the two cohorts, the diagnosis of NAFLD in the US population was mainly based on elevated ALT, after excluding viral hepatitis and alcoholic hepatitis, whereas the diagnosis of NAFLD in our study was based on ultrasonography. Studies report that 60% of the patients with NAFLD had normal ALT (50). This may be the reason for the high incidence of NAFLD in our study. Furthermore, since there are regional differences in NAFLD prevalence (53), there may be differences in NAFLD incidence in China and the United States. The report shows that NAFLD incidence in China has reached 20.9%, and the number of NAFLD patients currently exceeds 200 million (54). This is basically consistent with our research.

A cross-sectional study from South Korea, including 819 CKD patients, suggested that eGFR was positively associated with NAFLD after adjustment for relevant confounders (OR = 3.538, 95%CI: 1.801–6.948) (55). The reasons why their findings are inconsistent with ours may include the following: (1) The study population was different. Their study mainly focused on CKD patients in South Korea, while our study focused on Chinese people undergoing health check-ups in hospitals (2). The study design and the regression analysis methods used to explore the relationship between eGFR and NAFLD were different. They did not examine the non-linear relationship between the two. (3) Compared with our research, those studies did not consider the effect of DBP, BUN, HDL-c, SBP, and GGT on the relationship between eGFR and NAFLD when adjusting covariates. However, previous studies have identified these variables as factors associated with NAFLD or eGFR (43, 56–58). (4) This may be related to different renal functions. Several studies suggest that the association of eGFR and IR differs between CKD stages (59, 60). At the same time, insulin resistance is central to developing NAFLD (61). Concurrently, the sensitivity analysis found that the relationship remained stable across participants without excluding FPG > 6.1 mmol/L, ALT > 40 U/L, TG ≥ 1.7 mmol/L, or eGFR < 60 mL/min⋅1.73 m^2^. The efforts as mentioned above have confirmed the relationship’s stability between eGFR and NAFLD risk. The results provided a reference for clinical intervention in eGFR levels to reduce the risk of NAFLD.

Although there is no research on the relationship between eGFR and NAFLD in the Chinese non-obese hospital health check-up population. A recent study showed that lower eGFR is associated with an increased probability of liver fibrosis in Chinese diabetic and NAFLD patients. The study also found an inverse relationship between eGFR and insulin resistance (29). The underlying mechanism of the association between eGFR and NAFLD is still uncertain, but insulin resistance may be involved in the association. Studies have also confirmed the interaction between NAFLD and insulin resistance (62). Therefore, we propose that eGFR may affect the development of NAFLD by mediating insulin resistance. Besides, since there are no Food and Drug Administration-approved drugs to treat NAFLD, current treatment options include dietary restrictions and lifestyle changes. NAFLD is closely associated with metabolic disorders such as obesity, type 2 diabetes, and dyslipidemia. Hence, clinically various pharmacological approaches using existing drugs such as anti-diabetic, anti-obesity, antioxidants, and cytoprotective agents have been considered in managing NAFLD and NASH. However, several pharmacological therapies aiming to alleviate NAFLD-NASH are currently being examined at various phases of clinical trials (63). Our study found that eGFR was negatively associated with the risk of developing NAFLD. That is, as eGFR (renal function) declines, the risk of NAFLD increases accordingly. Therefore, clinically, for patients with chronic kidney disease (CKD), related treatment to delay renal function decline can effectively reduce the risk of NAFLD. At the same time, NAFLD also increases CKD risk (64). Active intervention can also reduce the risk of CKD and delay the further decline of renal function. As the most widely used index of obesity, BMI was revealed to be correlated with reduced eGFR, CKD, and end-stage renal disease (ESRD) (65, 66). Diabetes is the most common cause of CKD, and control of diabetes and its associated vascular complications can ameliorate CKD progression (67). In CKD, oxidative stress occurs frequently and has been proposed to be a central mechanism in the pathogenesis of CKD progression and CKD-associated complications and mortality (68). Therefore, the current anti-diabetic, anti-obesity, and antioxidant treatments for NAFLD can also delay the progression of CKD and reduce the risk of NAFLD. In addition, liraglutide, a GLP-1 receptor agonist, also demonstrated efficacy in reducing liver fat content as well as levels of liver enzymes in patients with NASH (69). SGLT2 has been reported to reduce hepatocyte injury biomarkers, improve liver steatosis, attenuate liver fibrosis, and improve liver function parameters (70–72). The study found that SGLT2 inhibitors reduce eGFR decline in subjects with well-regulated diabetes mellitus, pre-diabetes, or even non-diabetic CKD (73). Studies have also confirmed the renal protective effect of liraglutide (74, 75). The above studies suggest that NAFLD and CKD may have common molecular targets in terms of intervention. Protecting renal function may be a new therapeutic direction to reduce NAFLD risk.

In subgroup analysis, we found that FPG, HDL-c, ALT, and DBP could serve as the potential effect modifiers to modify the relationship between eGFR and NAFLD risk. Stronger associations were observed in the population with FPG ≤ 6.1 mmol/L, HDL-c < 1 mmol/L, ALT > 40 mmol/L, and DBP < 90 mmHg. In comparison, significantly weaker associations were detected in the people with DBP ≥ 90 mmHg, ALT < 40 U/L, FPG > 6.1 mmol/L, and HDL-c ≥ 1 mmol/L. Hypertension has been shown to be a risk factor for NAFLD (56), and HDL-c is negatively associated with the risk of NAFLD (76). Therefore, it is not surprising that the association of eGFR with NAFLD in the population with DBP ≥ 90 mmHg and HDL-c ≥ 1 mmol/L is weakened by the influence of DPB and HDL themselves on the risk of NAFLD. Although FPG and ALT are also closely associated with NAFLD risk (7, 48, 50), hyperglycemia is related to glomerular hyperperfusion and hyperfiltration (77). Our data analysis found that the proportion of eGFR > 130 mL/min/1.73 m^4^ was higher in participants with ALT ≤ 40 U/L. This may explain the weaker association between eGFR and NAFLD in the population with ALT < 40 U/L and FPG > 6.1 mmol/L.Since these factors could modify the relationship between eGFR and NAFLD, it is clinically possible to reduce the risk of NAFLD by altering the strength of the association between the eGFR and NAFLD by interfering with HDL-c, ALT, DBP, and FPG levels.

Furthermore, to the best of our knowledge, the present study observed a non-linear relationship between eGFR and NAFLD risk for the first time. The current study used a two-piecewise Cox proportional hazards regression model to clarify a non-linear relation between the eGFR and NAFLD risk. The inflection point was 103 mL/min/1.73 m^2^ after adjusting for confounders. It showed that when eGFR was below 103 mL/min/1.73 m^2^, a 1 unit decrease in the eGFR level was associated with a 1.2% greater adjusted HR of the risk of NAFLD (HR = 0.988, 95%CI: 0.984–0.991). However, When eGFR was in the range of 103–130 mL/min/1.73 m^2^, a 1 unit decrease in eGFR level was associated with a 3% greater adjusted HR of the risk NAFLD (HR = 0.971, 95%CI: 0.963–0.979). The reason is that other variables in the participants’ baseline may also have influenced NAFLD risk. It could be found that compared with the eGFR > 103 mL/min/1.73 m^2^ group, people with eGFR ≤ 103 mL/min/1.73 m^2^ have generally higher levels of BMI, ALT, BUN, TC, AST, TG, GGT, LDL-c, UA, SBP and DBP, and lower HDL-c levels (Supplementary Table 2). However, the above indicators were closely related to NAFLD (7, 43, 48–50, 56–58, 78, 79). When eGFR was less than 103 mL/min/1.73 m^2^, due to the presence of these NAFLD risk factors, eGFR had a relatively weak effect on NAFLD. On the contrary, when eGFR was greater than 103 mL/min/1.73 m^2^, the level of the risk factors for NAFLD, such as BMI, SBP, GGT, TG, BUN, AST, TC, ALT, LDL-c was lower, and the impact on NAFLD was weakened, at this time the effect of eGFR was relatively enhanced. It should be pointed out that when eGFR > 130 mL/min/1.73 m^2^, with the increase of eGFR, the risk of NAFLD no longer decreases accordingly. This may be related to early renal impairment manifested as glomerular hyperfiltration. Our findings provide an essential rationale for preventing NAFLD by intervening in the eGFR level in the clinic. When the eGFR level is in the range of 103–130 mL/min/1.73 m^2^, there is a significant negative association between eGFR and NAFLD risk. This study provides a reference for preventing NAFLD in people with different renal function statuses in the future. From a therapeutic perspective, it makes sense to maintain eGFR levels above the inflection point and below 130 mL/min/1.73 m^2^. Therefore, this assay has excellent clinical value. The findings of this research should be conducive to future studies on establishing a predictive model of NAFLD risk.

Our study has some strengths of not, and we listed them as follows: (1) A strength of our study is that the total sample size was relatively large. (2) To the best of our knowledge, this is the first time Chinese non-obese people have been used as a research population to explore the relationship between eGFR and NAFLD. (3) Information on covariates is complete and rarely missing. (4) This study explores non-linearity and explains them further. This is a very significant improvement over previous studies. (5) We used multiple imputations to handle missing data in this study. Multiple imputations can maximize statistical power and minimize potential bias caused by covariate information missing. (6) In this study, we ensured the robustness of the results through a series of sensitivity analyses (conversion of target-independent variable form, subgroup analysis, using a GAM to insert the continuity covariate into the equation as a curve, calculating E-values to explore the potential for unmeasured confounding, and reanalyzing the association between eGFR and NAFLD on individuals with complete data, or after excluding participants FPG > 6.1 mmol/L, ALT > 40 U/L, eGFR < 60 mL/min/1.73 m^2^, and TG ≥ 1.7 mmol/L). This makes our results more reliable.

Our research has the following shortcomings and needs attention: First, the study’s design is an observational study, so we cannot get the exact causal relationship because of the nature of the observational study design. Second, the findings can be generalized to Chinese non-obese people with a normal range of LDL-c only. The relationship of eGFR on NAFLD might be different in participants with BMI > 25 or LDL-c > 3.12 mmol/L. In the future, we can consider designing our studies and collecting all the participants, including normal weight and obese patients, with normal and abnormal LDL-c levels. Therefore, we can explore the relationship between the eGFR and NAFLD at different BMI and LDL-c levels. Third, as with all observational studies, although known potential confounders such as BMI, ALT, and TG were controlled, there may still be uncontrolled or unmeasured confounders. However, the authors calculated the E-value to quantify the potential impact of unmeasured confounders and found that unmeasured confounders were unlikely to explain the results. Fourth, a non-linear relationship is a type of relationship between two variables in which change in one entity does not correspond with constant change in the other variable. This might mean the relationship between the two variables seems unpredictable or virtually absent. However, non-linear entities can be related to each other in ways that are fairly predictable, but simply more complex than in a linear relationship. Because of the complexity of NAFLD pathogenesis, the relationship between eGFR and NAFLD is also complex, so the non-linear relationship may be closer to the actual relationship between eGFR and NAFLD. Finally, in this study, the diagnosis of NAFLD was made by ultrasonography rather than biopsy. This might have reduced the accuracy of the results. Furthermore, ultrasonography cannot differentiate steatosis from steatohepatitis. However, ultrasonography used to diagnose NAFLD has been widely used in epidemiological studies (80).

## Conclusion

This study demonstrates a negative and non-linear relationship between eGFR and NAFLD in Chinese non-obese people with a normal range of low-density lipoprotein cholesterol. There is a threshold effect between the eGFR level and NAFLD. When the eGFR level is in the range of 103–130 mL/min/1.73 m^2^, there is a significant negative association between eGFR and NAFLD risk. This study provides a reference for the prevention of NAFLD in people with different renal function statuses in the future. From a therapeutic perspective, it makes sense to maintain eGFR levels above the inflection point and below 130 mL/min/1.73 m^2^. Protecting renal function may be a new therapeutic direction to reduce NAFLD risk.

## Data Availability Statement

The original contributions presented in the study are included in the article/Supplementary Material, further inquiries can be directed to the corresponding author.

## Ethics Statement

The studies involving human participants were reviewed and approved by the Ethics Committee of Wenzhou People’s Hospital. The patients/participants provided their written informed consent to participate in this study.

## Author Contributions

JC, YH, YL, and HH conceived the research, drafted the manuscript, and did the statistical analysis. HH revised the manuscript and designed the study. All authors read and approved the final manuscript.

## Conflict of Interest

The authors declare that the research was conducted in the absence of any commercial or financial relationships that could be construed as a potential conflict of interest.

## Publisher’s Note

All claims expressed in this article are solely those of the authors and do not necessarily represent those of their affiliated organizations, or those of the publisher, the editors and the reviewers. Any product that may be evaluated in this article, or claim that may be made by its manufacturer, is not guaranteed or endorsed by the publisher.

## Footnotes

1
www.datadryad.org

2
http://www.R-project.org

3
http://www.empowerstats.com

4
https://doi.org/10.5061/dryad.1n6c4

## Abbreviations

NAFLD: Non-alcoholic fatty liver disease
eGFR: Evaluated glomerular filtration rate
FPG: Fasting plasma glucose
SBP: systolic blood pressure
NASH: non-alcoholic steatohepatitis
BMI: Body mass index
Scr: Serum creatinine
DBP: diastolic blood pressure
ALT: Alanine aminotransferase
UA: Uric acid
TC: Total cholesterol
TB: Total bilirubin
BUN: Serum urea nitrogen
ALP: Alkaline phosphatase
TG: Triglyceride
CKD-EPI: Chronic Kidney Disease Epidemiology Collaboration
ALB: Albumin
CKD: chronic kidney disease
ESRD: end-stage renal disease
AST: Aspartate aminotransferase
LDL-c: Low-density lipoprotein cholesterol
DBIL: Direct bilirubin
HDL-c: High-density lipoprotein cholesterol
GLB: Globulin
GGT: γ -Glutamyl transpeptidase
HR: Hazard ratio
Ref: Reference
CI: Confidence intervals
GAM: Generalized additive model
CKD: Chronic kidney disease
WHO: World Health Organization
HTG: Hypertriglyceridemia
MAR: missing-at-random
IFG: Impaired fasting glucose
IR: Insulin resistance
SD: Standard deviation.
