# Supplementary material for: Evaluated Glomerular Filtration Rate Is Associated With Non-alcoholic Fatty Liver Disease: A 5-Year Longitudinal Cohort Study in Chinese Non-obese People
Source: Front Nutr. 2022 Jun 16;9:916704. doi: 10.3389/fnut.2022.916704 (PMC9244698; doi:10.3389/fnut.2022.916704)
Supplement: Supplementary file 1 [file Data_Sheet_1.docx]

**Evaluated glomerular filtration rate is associated with non-alcoholic fatty liver disease: a 5-year longitudinal cohort study in Chinese non-obese people**

**Running title:** The association between eGFR and NAFLD

**Ji Cen^#1^, Yong Han^#2,3,8^, Yufei Liu^#4,5,8^, Haofei Hu****^*6,7,8^**

^1^Department of Nephrology, Hechi People's Hospital, Hechi 547000, Guangxi Zhuang Autonomous Region, China

^2^Department of Emergency, Shenzhen Second People’s Hospital, Shenzhen 518000, Guangdong Province, China

^3^Department of Emergency, The First Affiliated Hospital of Shenzhen University, Shenzhen 518000, Guangdong Province, China

^4^Department of Neurosurgery, Shenzhen Second People’s Hospital, Shenzhen 518000, Guangdong Province, China

^5^Department of Neurosurgery, The First Affiliated Hospital of Shenzhen University, Shenzhen 518000, Guangdong Province, China

^6^Department of Nephrology, Shenzhen Second People’s Hospital, Shenzhen 518000, Guangdong Province, China

^7^Department of Nephrology, The First Affiliated Hospital of Shenzhen University, Shenzhen 518000, Guangdong Province, China

^8^Shenzhen University Health Science Center, Shenzhen 518000, Guangdong Province, China

**^#^Ji Cen, Yong Han, and Yufei Liu have contributed equally to this work.**

***Corresponding author**

**Haofei HU**

Department of Nephrology,

Shenzhen Second People’s Hospital,

No.3002 Sungang Road, Futian District,

Shenzhen 518000,

Guangdong Province,

China

Tel:+86-755-83366388

E-mail: [huhaofei0319@126.com](mailto:huhaofei0319@126.com)

**Table S1. Collinearity diagnostics steps.**

| Variable | VIF  Step 1 | Step 2 |
| --- | --- | --- |
|  |  |  |
| Gender | 1 | 1 |
| Age(years) | 1.1 | 1.1 |
| ALP(U/L) | 1.2 | 1.2 |
| GGT(U/L) | 1.4 | 1.4 |
| ALT(U/L) | 3.1 | 3.1 |
| AST(U/L) | 3.3 | 3.3 |
| ALB(g/L) | 1.2 | 1.2 |
| GLB(g/L) | 1.1 | 1.1 |
| TB(umol/L) | 2 | 2 |
| DBIL(umol/L) | 2.1 | 2.1 |
| BUN(mmol/L) | 1.2 | 1.2 |
| UA(umol/L) | 1.5 | 1.5 |
| FPG(mmol/L) | 1.2 | 1.2 |
| TC(mmol/L) | 5.7 | NA |
| TG(mmol/L) | 2.1 | 1.5 |
| HDL-c(mmol/L) | 2.2 | 1.3 |
| LDL-c(mmol/L) | 4.4 | 1.1 |
| BMI(kg/m^2^) | 1.3 | 1.3 |
| SBP(mmHg) | 2.5 | 2.5 |
| DBP(mmHg) | 2.2 | 2.2 |

BMI, Body mass index; SBP, Systolic blood pressure; DBP, Diastolic blood pressure; ALP, Alkaline phosphatase; GGT, *γ*-glutamyl transpeptidase; ALT, Alanine aminotransferase; AST, Aspartate aminotransferase; ALB, albumin; GLB, globulin; TC, Total cholesterol; TG, Triglyceride; HDL-c, High-density lipoprotein cholesterol; LDL-c, Low-density lipid cholesterol; BUN, Serum urea nitrogen; Scr, Serum creatinine; FPG, Fasting plasma glucose; UA, uric acid; DBIL, Direct bilirubin; TB, Total bilirubin

Abbreviation: VIF: variance inflation factor; VIF = 1/(1-R^2^).

Note: The variables with VIF>5 will be regarded as collinear variables and cannot be included in the multiple regression model.

**Table S2 Relationship between eGFR and the incident NAFLD for individuals with complete data**

| Exposure | Crude model (HR,95%CI,P) | Model I(HR,95%CI,P) | Model II(HR,95%CI,P) |
| --- | --- | --- | --- |
| eGFR | 0.980 (0.978, 0.981) <0.00001 | 0.985 (0.983, 0.987) <0.00001 | 0.994 (0.990, 0.998) 0.00162 |
| eGFR Quartile |  |  |  |
| Q1 | 1.0 | 1.0 | 1.0 |
| Q2 | 0.697 (0.630, 0.771) <0.00001 | 0.765 (0.689, 0.848) <0.00001 | 0.876 (0.757, 1.013) 0.07424 |
| Q3 | 0.464 (0.415, 0.519) <0.00001 | 0.608 (0.540, 0.685) <0.00001 | 0.745 (0.621, 0.894) 0.00155 |
| Q4 | 0.238 (0.208, 0.272) <0.00001 | 0.364 (0.314, 0.423) <0.00001 | 0.730 (0.565, 0.943) 0.01586 |
| P for trend | <0.00001 | <0.00001 | 0.00105 |

Crude model: we did not adjust other covariants(N=16138)

Model I: we adjusted age, DBP, sex, BMI, SBP(N=16118)

Model II: we adjusted age, SBP, sex, ALT, BMI, GGT, DBP, ALP, ALB, HDL-c, GLB, DBIL, AST, TB, UA, FBG, TG, BUN, LDL-c(N=6132)

HR, Hazard ratios; CI: confidence, Ref: reference; eGFR, evaluated glomerular filtration rate(mL/min·1.73 m2); NAFLD, non-alcoholic fatty liver disease

**TableS3. The characteristics of participants on both sides of the inflection point.**

| eGFR group | <103.489 | >=103.489 | P-value |
| --- | --- | --- | --- |
| Participants | 9061 | 7077 |  |
| Age(years) | 47.66 ± 16.21 | 37.52 ± 10.75 | <0.001 |
| BMI(kg/m^2^) | 21.76 ± 2.01 | 20.90 ± 2.00 | <0.001 |
| SBP(mmHg) | 123.96 ± 17.02 | 116.44 ± 15.16 | <0.001 |
| DBP(mmHg) | 74.36 ± 10.36 | 70.81 ± 9.99 | <0.001 |
| TC(mmol/L) | 4.66 ± 0.74 | 4.58 ± 0.74 | <0.001 |
| TG(mmol/L) | 1.18 (0.88-1.65) | 0.96 (0.72-1.33) | <0.001 |
| HDL-c(mmol/L) | 1.44 ± 0.36 | 1.49 ± 0.37 | <0.001 |
| LDL-c(mmol/L) | 2.31 ± 0.47 | 2.21 ± 0.46 | <0.001 |
| FPG(mmol/L) | 5.24 ± 0.85 | 5.02 ± 0.66 | <0.001 |
| UA(umol/L) | 306.92 ± 85.51 | 244.66 ± 72.33 | <0.001 |
| Scr(umol/L) | 88.26 ± 16.89 | 64.55 ± 10.92 | <0.001 |
| eGFR(mL/min·1.73 m^2^) | 82.40 ± 14.68 | 119.87 ± 11.02 | <0.001 |
| BUN(mmol/L) | 4.77 ± 1.34 | 4.27 ± 1.15 | <0.001 |
| ALP(U/L) | 73.91 ± 23.47 | 67.57 ± 21.88 | <0.001 |
| GGT(U/L) | 23.00 (17.00-35.00) | 19.00 (13.00-32.00) | <0.001 |
| ALT(U/L) | 17.00 (13.00-25.00) | 16.00 (11.00-24.00) | <0.001 |
| AST(U/L) | 23.71 ± 9.80 | 21.84 ± 8.90 | <0.001 |
| ALB(g/L) | 44.49 ± 2.79 | 44.32 ± 2.60 | <0.001 |
| GLB(g/L) | 29.44 ± 3.99 | 29.55 ± 3.68 | 0.059 |
| TB(umol/L) | 12.53 ± 4.98 | 11.64 ± 4.94 | <0.001 |
| DBIL(umol/L) | 2.20 (1.50-3.00) | 2.10 (1.44-2.91) | 0.003 |
| Gender |  |  | <0.001 |
| Female | 4972 (54.87%) | 2699 (38.14%) |  |
| Male | 4089 (45.13%) | 4378 (61.86%) |  |

Values are n (%) or mean ± SD or median (quartile)

BMI, Body mass index; SBP, Systolic blood pressure; DBP, Diastolic blood pressure; ALP, Alkaline phosphatase; GGT, *γ*-glutamyl transpeptidase; ALT, Alanine aminotransferase; AST, Aspartate aminotransferase; ALB, albumin; GLB, globulin; TC, Total cholesterol; TG, Triglyceride; HDL-C, High-density lipoprotein cholesterol; LDL-C, Low-density lipid cholesterol; BUN, Serum urea nitrogen; Scr, Serum creatinine; FPG, Fasting plasma glucose; UA, uric acid; eGFR, evaluated glomerular filtration rate; DBIL, Direct bilirubin; TB, Total bilirubin

**Figure S1. Levels of eGFR of all participants from the NAFLD and non-NAFLD groups..**

Figure S1 indicated that the level of eGFR in the NAFLD group was lower. In contrast, the eGFR level in the non-NAFLD group was relatively higher.
