# Supplementary figures and images for: Evaluated Glomerular Filtration Rate Is Associated With Non-alcoholic Fatty Liver Disease: A 5-Year Longitudinal Cohort Study in Chinese Non-obese People
Source: Front Nutr. 2022 Jun 16;9:916704. doi: 10.3389/fnut.2022.916704 (PMC9244698; doi:10.3389/fnut.2022.916704)

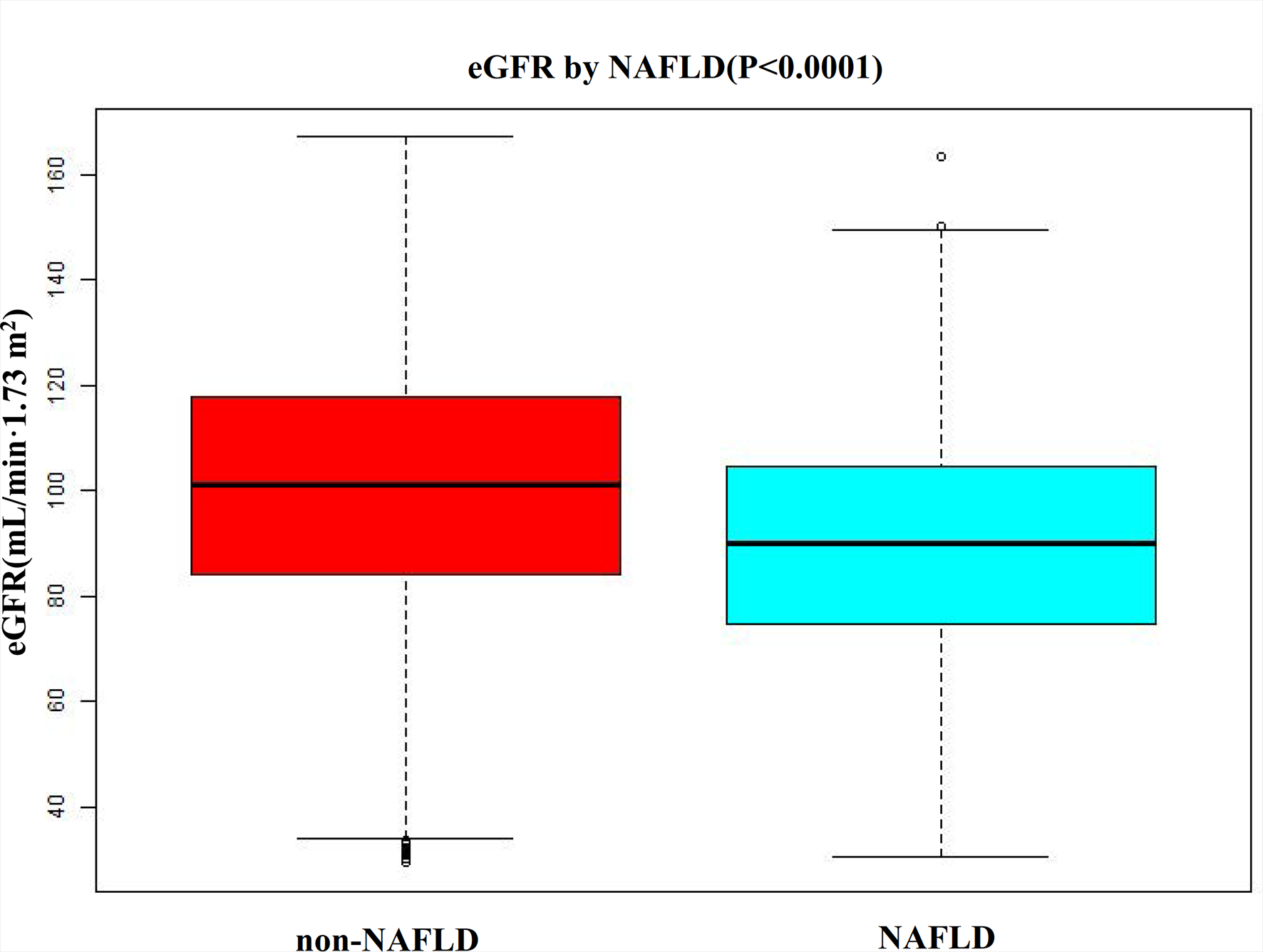

Supplement: Supplementary file 3 [file Image_1.TIF]
